# Effectiveness of two and three mRNA COVID‐19 vaccine doses against Omicron‐ and Delta‐Related outpatient illness among adults, October 2021–February 2022

**DOI:** 10.1111/irv.13029

**Published:** 2022-07-29

**Authors:** Sara S. Kim, Jessie R. Chung, H. Keipp Talbot, Carlos G. Grijalva, Karen J. Wernli, Erika Kiniry, Emily T. Martin, Arnold S. Monto, Edward A. Belongia, Huong Q. McLean, Manjusha Gaglani, Mufaddal Mamawala, Mary Patricia Nowalk, Krissy Moehling Geffel, Sara Y. Tartof, Ana Florea, Justin S. Lee, Mark W. Tenforde, Manish M. Patel, Brendan Flannery, Strain Surveillance and Emerging Variants Team, Meghan L. Bentz, Alex Burgin, Mark Burroughs, Morgan L. Davis, Dakota Howard, Kristine Lacek, Joseph C. Madden, Sarah Nobles, Jasmine Padilla, Mili Sheth

**Affiliations:** ^1^ Centers for Disease Control and Prevention Atlanta Georgia USA; ^2^ Vanderbilt University Medical Center Nashville Tennessee USA; ^3^ Kaiser Permanente Washington Health Research Institute Seattle Washington USA; ^4^ University of Michigan School of Public Health Ann Arbor Michigan USA; ^5^ Marshfield Clinic Research Institute Marshfield Wisconsin USA; ^6^ Baylor Scott and White Health Temple Texas USA; ^7^ Texas A&M University College of Medicine Temple Texas USA; ^8^ University of Pittsburgh Schools of Health Sciences Pittsburgh Pennsylvania USA; ^9^ Kaiser Permanente Southern California Pasadena California USA

**Keywords:** COVID‐19, outpatient, vaccine effectiveness

## Abstract

**Background:**

We estimated SARS‐CoV‐2 Delta‐ and Omicron‐specific effectiveness of two and three mRNA COVID‐19 vaccine doses in adults against symptomatic illness in US outpatient settings.

**Methods:**

Between October 1, 2021, and February 12, 2022, research staff consented and enrolled eligible participants who had fever, cough, or loss of taste or smell and sought outpatient medical care or clinical SARS‐CoV‐2 testing within 10 days of illness onset. Using the test‐negative design, we compared the odds of receiving two or three mRNA COVID‐19 vaccine doses among SARS‐CoV‐2 cases versus controls using logistic regression. Regression models were adjusted for study site, age, onset week, and prior SARS‐CoV‐2 infection. Vaccine effectiveness (VE) was calculated as (1 − adjusted odds ratio) × 100%.

**Results:**

Among 3847 participants included for analysis, 574 (32%) of 1775 tested positive for SARS‐CoV‐2 during the Delta predominant period and 1006 (56%) of 1794 participants tested positive during the Omicron predominant period. When Delta predominated, VE against symptomatic illness in outpatient settings was 63% (95% CI: 51% to 72%) among mRNA two‐dose recipients and 96% (95% CI: 93% to 98%) for three‐dose recipients. When Omicron predominated, VE was 21% (95% CI: −6% to 41%) among two‐dose recipients and 62% (95% CI: 48% to 72%) among three‐dose recipients.

**Conclusions:**

In this adult population, three mRNA COVID‐19 vaccine doses provided substantial protection against symptomatic illness in outpatient settings when the Omicron variant became the predominant cause of COVID‐19 in the United States. These findings support the recommendation for a third mRNA COVID‐19 vaccine dose.

## 1 BACKGROUND

On November 29, 2021, the Centers for Disease Control and Prevention (CDC) recommended that all adults aged ≥18 years receive a third mRNA COVID‐19 vaccine booster dose at least 6 months after completing a two‐dose primary series.^1^ The 6‐month interval recommendation was shortened to at least 5 months on January 4, 2022, for the Pfizer‐BioNTech vaccine and on January 7, 2022, for the Moderna vaccine. These recommendations were released during the emergence of the SARS‐CoV‐2 Omicron variant, which was first detected in the United States on December 1, 2021.^2^ Effectiveness of two mRNA vaccine doses against symptomatic illness or hospitalization due to infection with the Omicron variant has been lower compared with the Delta variant, with increased protection against both variants after receipt of a third dose.^3^, ^4^, ^5^ However, data comparing two‐ and three‐dose vaccine effectiveness (VE) against symptomatic COVID‐19 in outpatient settings during periods when the Delta and Omicron variants predominated are limited, especially among COVID‐19 cases identified through active surveillance where all enrolled participants with COVID‐19‐like illness (CLI) are tested for SARS‐CoV‐2.

Studies with active enrollment such as the US Flu Vaccine Effectiveness Network (US Flu VE Network) provide access to specimens for research purposes including whole genome sequencing and access to data not available in medical records including risk factors for SARS‐CoV‐2 infection.^6^ To assess the impact of a third dose in the context of emerging variants with immune evasion^7^ and potential waning immunity, we estimated variant‐specific effectiveness of two versus three mRNA vaccine doses against symptomatic illness in outpatient settings. Additionally, we utilized virus sequencing data to define periods when Delta and Omicron variants each predominated.

## 2 METHODS

### 2.1 Study design and population

This study was conducted within the US Flu VE Network, which consists of participating health systems in seven states: California, Michigan, Pennsylvania, Tennessee, Texas, Washington, and Wisconsin. Between October 1, 2021, and February 12, 2022, research staff screened patients seeking outpatient medical care or SARS‐CoV‐2 clinical testing with acute respiratory infection (ARI).^8^ Eligible participants reported onset of fever, cough, or loss of taste/smell with symptom duration of <10 days^8^ and had a clinical or research respiratory specimen collected for SARS‐CoV‐2 molecular testing within 10 days of illness onset. Research staff consented and enrolled eligible participants, who may have sought in‐person medical care for ARI, completed a telehealth visit, or sought SARS‐CoV‐2 testing. Enrolled participants completed surveys with standardized questions across all research sites at enrollment including questions about demographics, symptoms experienced for current illness, COVID‐19 vaccination history, prior SARS‐CoV‐2 infection, general health status, and high‐risk SARS‐CoV‐2 exposures (healthcare worker with close patient contact; contact with another laboratory‐confirmed SARS‐CoV‐2 case in the 14 days before illness onset; or household member with laboratory‐confirmed SARS‐CoV‐2 or with symptoms consistent with COVID‐19, i.e., cough, fever, chills, or loss of taste or smell, in the 14 days before illness onset). Participants were asked broadly whether they have any serious chronic medical condition such as heart disease, lung disease, diabetes, cancer, liver or kidney disease, immune suppression, or high blood pressure. Information about individual conditions, including severity, was not available. This activity was reviewed and approved by the CDC and each US Flu VE Network site's Institutional Review Board. *

### 2.2 SARS‐CoV‐2 status

Participants were tested for SARS‐CoV‐2 by reverse‐transcription polymerase chain reaction tests using respiratory specimens collected for clinical or research purposes. We classified participants with a positive SARS‐CoV‐2 result as cases. Participants who had discordant clinical and research results were categorized as a case if at least one of the results were positive. We classified participants with only negative SARS‐CoV‐2 results as controls.

In addition, SARS‐CoV‐2 virus variants from a subset of SARS‐CoV‐2 positive participants with onset dates between November 9, 2021, and January 9, 2022, were identified by whole genome sequencing. Research‐collected SARS‐CoV‐2 positive respiratory specimens with cycle threshold values <30 and stored in appropriate transport medium were prepared for sequencing using the xGen SARS‐CoV‐2 library preparation kit (Integrated DNA Technologies, Inc., Coralville, IA). Libraries were sequenced on a NovaSeq instrument (Illumina Inc., San Diego, CA). A single consensus genome for each sample was generated. SARS‐CoV‐2 variants were determined using Pangolin version 3.1.20 (pangoLEARN 1.2.123, Scorpio 0.3.16).^9^

### 2.3 COVID‐19 vaccination status

COVID‐19 vaccination status was verified using electronic medical records, immunization information systems, and vaccination record cards. Participants considered vaccinated with two doses were those who received two mRNA vaccine doses ≥14 days before illness onset (two‐dose). To be considered for the two‐dose analyses, participants must have received doses ≥16 days apart for Pfizer‐BioNTech vaccines and ≥23 days apart for Moderna vaccines. Participants considered vaccinated with three doses were those who received three mRNA vaccine doses, where the third dose was given ≥7 days before illness onset (three‐dose).^4^ Participants who received a third dose before the recommended ≥150 days after the second dose were also considered three‐dose recipients but excluded from sensitivity analyses. Three‐dose recipients included both immunocompromised participants who received a third dose as a primary series and otherwise healthy participants who received a third dose as a booster. Those who did not report vaccine receipt and had no documentation of an mRNA COVID‐19 vaccination before illness onset were defined as unvaccinated. We excluded participants who self‐reported COVID‐19 vaccination but were missing verified documentation of doses received.

### 2.4 Statistical analyses

We limited analyses to adults aged ≥18 years. Using the test‐negative design,^10^ we compared the odds of two‐ or three‐dose mRNA COVID‐19 vaccination among COVID‐19 cases versus test‐negative controls using logistic regression. VE was calculated as (1 − adjusted odds ratio) × 100%. Regression models were adjusted for variables identified a priori including study site, age, and illness onset week. Sex, race and ethnicity, illness onset to specimen collection interval, self‐reported high‐risk exposure, self‐reported chronic medical condition, and self‐reported prior SARS‐CoV‐2 infection were evaluated as model covariates using a change‐in‐estimate (≥5% change in odds ratio) forward stepwise approach. In addition to covariates included a priori, prior SARS‐CoV‐2 infection was included in the final regression model because its inclusion changed the OR by 8%. All other potential confounders examined changed the OR by <1%.

We evaluated VE by variant, either sequence‐confirmed variant or using time periods of predominant Delta (illness onset of October 1–December 9, 2021) versus Omicron circulation (illness onset of December 20, 2021–February 12, 2022) when variant was not confirmed by sequencing. These periods were selected based on the SARS‐CoV‐2 sequencing results on a subset of cases in the US Flu VE Network. Due to co‐circulation of the Delta and Omicron variants between December 10 and 19, 2021, we excluded participants without sequenced viruses with onset dates during this period for variant‐specific estimates. We also assessed potential waning immunity among two‐dose recipients by comparing VE of those who received their second dose 14–149 days versus ≥150 days prior to illness onset during each variant predominant period.

We conducted several subgroup analyses where three‐dose VE was stratified by self‐reported high‐risk exposure status, self‐reported chronic medical condition, self‐reported prior SARS‐CoV‐2 infection, days between illness onset and specimen collection date, and self‐reported presence of fever with cough or shortness of breath during the Delta and Omicron predominant periods. Analyses by illness onset to specimen collection interval were performed to identify bias resulting from potential false negative SARS‐CoV‐2 test results among participants who presented for care or testing later than those presenting 0–2 days after illness onset.^10^ Analyses by symptoms were performed to evaluate VE among persons with potentially more severe illness compared to those without fever paired with cough or shortness of breath, indicating more mild illness.

## 3 RESULTS

### 3.1 Study population

**FIGURE 1:**
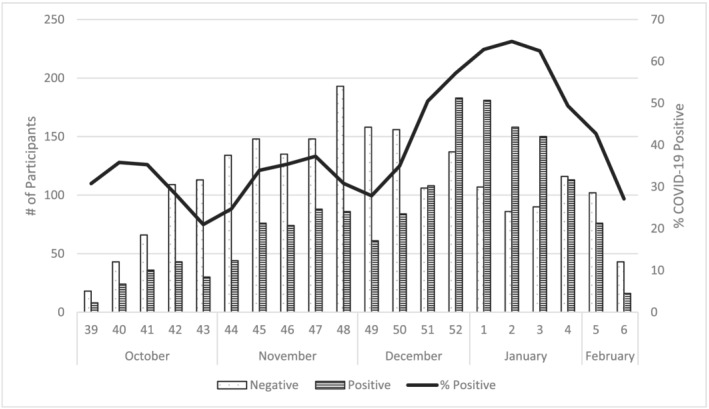
Number and percent of SARS‐CoV‐2 positive enrolled participants by onset week, US Flu VE Network, October 2021–February 2022

Between October 2021 and February 2022, US Flu VE Network sites enrolled 4448 eligible outpatients aged ≥18 years, among whom 601 were excluded due to receiving a non‐mRNA vaccine (n = 216), self‐reporting vaccination history with no documentation available (n = 145), receiving one mRNA COVID‐19 vaccine dose (n = 121), missing vaccine product information (n = 55), missing SARS‐CoV‐2 testing information (n = 34), or having an indeterminate vaccination status (n = 30). Among 3847 included for analysis, 575 (32%) of 1775 participants tested SARS‐CoV‐2 positive during the Delta predominant period and 1006 (56%) of 1794 participants tested positive during the Omicron predominant period. There were 278 participants whose illness onset dates fell between the defined Delta and Omicron predominance periods. SARS‐CoV‐2 positivity reached over 50% during the third week of December and peaked at 64% during the second week of January (Figure 1).

Over the entire study period, participants who self‐reported a high‐risk exposure or reported fever were more likely to test positive (Table 1). Additionally, participants who were aged ≥65 years, identified as White non‐Hispanic or other race non‐Hispanic compared with Black non‐Hispanic or Hispanic, self‐reported a chronic medical condition, and did not self‐report a fever were more likely to receive a third vaccine dose (Table 2). Among two‐dose recipients, the median interval between receipt of a second dose and illness onset date was 225 days (range 14–386); 13% and 87% had received a second mRNA vaccine dose 14–149 days or ≥150 days prior to illness onset, respectively (data not shown). The median interval between third dose receipt and illness onset was 53 days (range: 7–230) (data not shown).

**TABLE 1:** Characteristics of symptomatic adults seeking outpatient medical care or clinical SARS‐CoV‐2 testing by SARS‐CoV‐2 status, US Flu VE Network, October 2021–February 2022

	Negative SARS‐CoV‐2 Participants with CLI		Positive SARS‐CoV‐2 Participants with CLI		
	N	Col %	N	Col %	P value^a^
Total	2208	100	1639	100	
Age group, y					0.06
18–49	1347	61	1027	63	
50–64	544	25	419	26	
≥65	317	14	193	12	
Site					<0.01
California	322	15	164	10	
Michigan	166	8	188	11	
Pennsylvania	311	14	320	20	
Tennessee	269	12	186	11	
Texas	262	12	159	10	
Washington	296	13	110	7	
Wisconsin	582	26	512	31	
Sex^b^					<0.01
Female	1485	67	1017	62	
Male	720	33	618	38	
Race/ethnicity^c^					<0.01
Black, non‐Hispanic	87	4	119	7	
Hispanic	221	10	134	8	
Other, non‐Hispanic	198	9	128	8	
White, non‐Hispanic	1676	77	1218	76	
Self‐reported chronic medical condition^d^					0.77
No	1514	70	1138	70	
Yes	652	30	480	30	
High‐risk exposure					<0.01
No	1273	58	706	43	
Yes	935	42	933	57	
Self‐reported prior infection^e^					<0.01
No	1841	84	1450	89	
Yes (6 missing)	350	16	179	11	0.12
<3 months ago	194	56	110	63	
≥3 months ago	154	44	65	37	
Product among vaccinated (2 and 3 doses)					
Moderna	670	36	368	31	
Pfizer‐BioNTech	1165	62	784	67	
Combination	42	2	20	2	
Fever^f^					<0.01
No	1233	56	649	40	
Yes	950	44	968	60	

Abbreviation: CLI, COVID‐19‐like illness.

^a^ P value for chi‐square statistic.

^b^ Seven participants missing data on sex.

^c^ Sixty‐six participants missing data on race and/or ethnicity.

^d^ Sixty‐three participants missing data on chronic medical condition.

^e^ Twenty‐seven participants missing data on self‐reported prior SARS‐CoV‐2 infection.

^f^ Forty‐seven participants missing data on presence of fever.

**TABLE 2:** Characteristics of symptomatic adults seeking outpatient medical care or SARS‐CoV‐2 clinical testing by mRNA COVID‐19 verified vaccination status, US Flu VE Network, October 2021–February 2022

	Unvaccinated		2 doses (14–149 days)		2 doses (≥150 days)		3 doses		
	N	Row %	N	Row %	N	Row %	N	Row %	P value^a^
Total	798	21	256	7	1681	44	1112	29	
Age group, y									<0.01
18–49	566	24	185	8	1099	46	524	22	
50–64	181	19	63	7	399	41	320	33	
≥65	51	10	8	2	183	36	268	53	
Site									<0.01
California	15	3	24	5	268	55	179	37	
Michigan	50	14	15	4	183	52	106	30	
Pennsylvania	208	33	29	5	292	46	102	16	
Tennessee	56	12	44	10	194	43	161	35	
Texas	130	31	49	12	179	43	63	15	
Washington	12	3	23	6	210	52	161	40	
Wisconsin	327	30	72	7	355	32	340	31	
Sex^b^									0.09
Female	496	20	168	7	1086	43	752	30	
Male	302	23	88	7	589	44	359	27	
Race/ethnicity^c^									<0.01
Black, non‐Hispanic	61	30	27	13	76	37	42	20	
Hispanic	45	13	29	8	199	56	82	23	
Other, non‐Hispanic	32	10	16	5	170	52	108	33	
White, non‐Hispanic	646	22	180	6	1207	42	861	30	
Self‐reported chronic medical condition^d^									<0.01
No	584	22	193	7	1190	45	685	26	
Yes	205	18	61	5	461	41	405	36	
High‐risk exposure									<0.01
No	406	21	134	7	916	46	523	26	
Yes	392	21	122	7	765	41	589	32	
Self‐reported prior infection^e^									<0.01
No	626	19	198	6	1466	45	1001	30	
Yes (6 missing)	169	32	55	10	200	38	105	20	<0.01
<3 months ago	123	40	25	8	96	32	60	20	
≥3 months ago	45	21	29	13	101	46	44	20	
Product among vaccinated (2 and 3 doses)									
Moderna			64	6	636	61	338	33	
Pfizer‐BioNTech			190	10	1045	54	714	37	
Combination			2	3	0	0	60	97	
Fever^f^									<0.01
No	325	17	120	6	795	42	642	34	
Yes	468	24	135	7	862	45	453	24	

^a^ P value for chi‐square statistic.

^b^ Seven participants missing data on sex.

^c^ Sixty‐six participants missing data on race and/or ethnicity.

^d^ Sixty‐three participants missing data on chronic medical condition.

^e^ Twenty‐seven participants missing data on self‐reported prior SARS‐CoV‐2 infection.

^f^ Forty‐seven participants missing data on presence of fever.

### 3.2 Study periods by variant predominance

**FIGURE 2:**
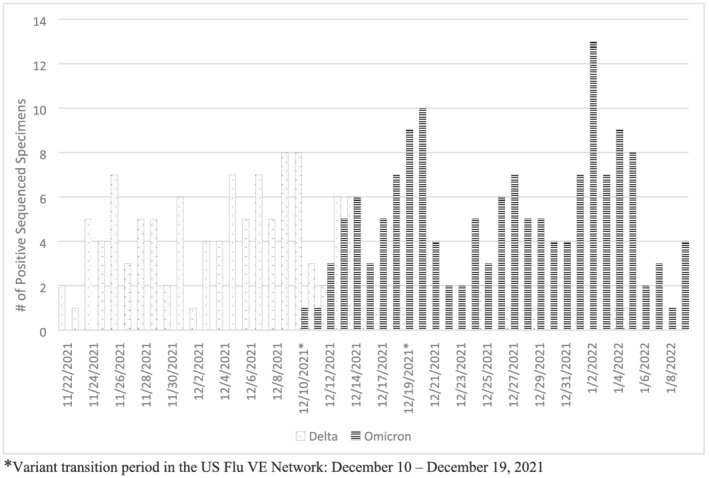
SARS‐CoV‐2 variant virus distribution among subset of symptomatic SARS‐CoV‐2 positive participants by onset date, November 2021–January 2022. *Variant transition period in the US Flu VE Network: December 10–December 19, 2021

Sequencing results from 272 out of 873 SARS‐CoV‐2‐positive US Flu VE Network participants with onset dates between November 9, 2021, and January 9, 2022, demonstrated distinct periods of Delta versus Omicron circulation with co‐circulation of both variants during December 10–19, 2021 (Figure 2). Overall, 45% of sequenced specimens were Delta. The first Omicron variant in the network was detected on December 10, 2021, and it became the consistently predominant variant (>50% of sequenced viruses) by December 15, 2021, with few viruses in early January 2022 still being identified as Delta.

### 3.3 Vaccine effectiveness

During the Delta period, adjusted VE against symptomatic illness in outpatient settings was 63% (95% CI: 51% to 72%) among mRNA two‐dose recipients and 96% (95% CI: 93% to 98%) for three‐dose recipients (Table 3). During the Omicron period, adjusted VE was 21% (95% CI: −6% to 41%) among two‐dose recipients and 62% (95% CI: 48% to 72%) among three‐dose recipients. During the Delta period, VE among participants who received their second dose 14–149 days before illness onset was 89% (95% CI: 78% to 94%) compared with 58% (95% CI: 44% to 68%) among those who received their second dose ≥150 days before illness onset (Table 3). During the Omicron period, VE among those who received their second dose 14–149 days before illness onset was 45% (14% to 66%) and among those who received their second dose ≥150 days before illness onset was 11% (−21% to 35%). Excluding three‐dose recipients who received the third dose <150 days after the second dose (n = 35) did not change three‐dose VE estimates (data not shown).

**TABLE 3:** Two‐ and three‐dose vaccine effectiveness during SARS‐CoV‐2 Delta variant or Omicron variant associated symptomatic COVID‐19 illness among adults seeking outpatient medical care or SARS‐CoV‐2 clinical testing, US Flu VE Network, October 2021–February 2022

	SARS‐CoV‐2 positive		SARS‐CoV‐2 negative		Unadjusted VE		Adjusted^a^ VE	
	Vaccinated/Total	(%)	Vaccinated/Total	(%)	VE	(95% CI)	VE	(95% CI)
Overall								
2‐dose	822/1289	(64)	1115/1446	(77)	48	(38 to 56)	48	(37 to 57)
3‐dose	350/817	(43)	762/1093	(70)	67	(61 to 73)	78	(72 to 83)
Delta^b^								
2‐dose	327/552	(59)	763/942	(81)	66	(57 to 73)	63	(51 to 72)
14–149 days	14/239	(6)	106/285	(37)	89	(81 to 94)	89	(78 to 94)
≥150 days	313/538	(58)	657/836	(79)	62	(52 to 70)	58	(44 to 68)
3‐dose	22/247	(9)	259/438	(59)	93	(89 to 96)	96	(93 to 98)
Omicron^b^								
2‐dose	464/684	(68)	257/380	(68)	0	(−32 to 23)	21	(−6 to 41)
14–149 days	69/289	(24)	53/176	(30)	27	(−11 to 52)	45	(14 to 66)
≥150 days	395/615	(64)	204/327	(62)	−8	(−43 to 18)	11	(−21 to 35)
3‐dose	322/542	(59)	408/531	(77)	56	(43 to 66)	62	(48 to 72)

^a^ Logistic regression model adjusted for age, site, illness onset week, and prior infection status.

^b^ Totals in variant‐specific periods may not add up to overall total as a transition period was included in the overall estimates but removed in the variant‐specific periods.

### 3.4 Vaccine effectiveness by subgroup

Self‐reported high‐risk exposure status, self‐reported presence of a chronic medical condition, self‐reported prior laboratory‐confirmed SARS‐CoV‐2 infection, longer interval from illness onset to respiratory specimen collection, and self‐reported presence of fever with cough or shortness of breath did not change three‐dose VE during the Delta variant predominant period (Table 4). However, during the period when the Omicron variant predominated, three‐dose VE point estimates tended to be lower but with overlapping confidence intervals among those who had a high‐risk exposure, a chronic medical condition, a prior SARS‐CoV‐2 infection, or CLI that included fever. During the Delta period, 4% of cases and 14% of controls had prior infection compared with the Omicron period when 15% of cases and 20% of controls had prior infection.

**TABLE 4:** Results of subgroup analyses of three‐dose vaccine effectiveness against Delta and Omicron variant related symptomatic COVID‐19 illness

	SARS‐CoV‐2 positive		SARS‐CoV‐2 negative		Unadjusted VE		Adjusted^a^ VE	
	Vaccinated/Total	(%)	Vaccinated/Total	(%)	VE	(95% CI)	VE	(95% CI)
Delta								
High‐risk exposure								
No	9/107	(8)	145/261	(56)	93	(85 to 96)	98	(94 to 99)
Yes	13/140	(9)	114/177	(64)	94	(89 to 97)	96	(92 to 98)
Chronic medical condition								
No	7/161	(4)	134/259	(52)	96	(91 to 98)	98	(94 to 99)
Yes	15/84	(18)	120/168	(71)	91	(83 to 95)	95	(87 to 98)
Prior infection								
No	20/228	(9)	239/378	(63)	94	(91 to 97)	97	(95 to 99)
Yes	1/17	(6)	20/60	(33)	87	(−1 to 98)	79	(−81 to 98)
Days from illness onset to respiratory specimen collection								
0–2 days	17/206	(8)	212/355	(60)	94	(90 to 96)	97	(94 to 99)
3–10 days	5/41	(12)	47/83	(57)	89	(70 to 96)	93	(70 to 98)
Fever + cough/shortness of breath								
No	9/72	(13)	131/195	(67)	93	(85 to 97)	95	(86 to 98)
Yes	13/175	(7)	128/243	(53)	93	(87 to 96)	97	(94 to 99)
Omicron								
High‐risk exposure								
No	111/218	(51)	199/253	(79)	72	(58 to 81)	76	(61 to 86)
Yes	211/324	(65)	209/278	(75)	38	(12 to 57)	49	(23 to 66)
Chronic medical condition								
No	201/378	(53)	266/360	(74)	60	(45 to 71)	66	(50 to 76)
Yes	117/159	(74)	131/160	(82)	38	(−5 to 64)	45	(−4 to 71)
Prior infection								
No	291/460	(63)	356/428	(83)	65	(52 to 75)	64	(49 to 75)
Yes	29/79	(37)	49/99	(49)	41	(−8 to 68)	52	(−1 to 77)
Days from illness onset to respiratory specimen collection								
0–2 days	295/487	(61)	360/472	(76)	52	(37 to 64)	60	(44 to 71)
3–10 days	27/55	(49)	48/59	(81)	78	(49 to 90)	72	(18 to 91)
Fever + cough/shortness of breath								
No	142/215	(66)	221/261	(85)	65	(45 to 77)	68	(47 to 81)
Yes	180/327	(55)	187/270	(69)	46	(24 to 61)	55	(31 to 70)

^a^ Logistic regression model adjusted for age, site, illness onset week, and prior infection status.

## 4 DISCUSSION

This investigation adds to early evidence of effectiveness of a third mRNA vaccine dose against laboratory‐confirmed SARS‐CoV‐2 infection among adults seeking outpatient care and clinical testing for CLI symptoms during the pandemic wave predominated by the Omicron variant.^3^, ^11^, ^12^, ^13^, ^14^ However, three‐dose effectiveness among adults was lower during the Omicron predominant period than during the pandemic wave associated with the Delta variant. Similar to analyses of large electronic medical record databases or data from SARS‐CoV‐2 testing sites, three‐dose VE in this analysis was higher against Delta than against Omicron‐related illness.^3^, ^11^, ^12^

Findings from the US Flu VE Network are also consistent with higher estimates of two‐dose VE when the second dose was given less than 5 months before current illness onset compared with at least 5 months or more before illness onset.^3^, ^5^ Waning effectiveness against SARS‐CoV‐2 Delta variant virus infection or associated outpatient illness was also observed 5 to 6 months after receipt of the second mRNA vaccine dose in other countries using multiple study designs.^15^, ^16^, ^17^, ^18^, ^19^ However, among US Flu VE Network participants, two‐dose mRNA VE point estimate against outpatient illness associated with the Delta variant among those who received their second dose at least 5 months or more before illness onset remained higher than two‐dose VE against Omicron among those who received their second dose <5 months before illness onset, with overlapping 95% confidence intervals. These results suggest that updates to COVID‐19 vaccine formulations or additional booster doses may be needed to improve protection against future SARS‐CoV‐2 variant viruses.

Active enrollment of study participants in the US Flu VE Network provided additional information to evaluate differences in three‐dose mRNA VE according to participants' symptoms, reported history of past laboratory‐confirmed SARS‐CoV‐2 infection, high‐risk exposure, and presence of underlying medical conditions. First, among generally healthy outpatients with symptomatic illness enrolled in the US Flu VE Network, three‐dose VE point estimates during the Omicron period tended to be lower among participants reporting underlying medical conditions compared to point estimates among participants without underlying conditions. Presence of underlying medical conditions, especially immunosuppressive conditions, have been associated with decreased mRNA VE against severe outcomes including COVID‐19 related hospitalizations^3^, ^20^, ^21^, ^22^, ^23^, ^24^, ^25^, ^26^, ^27^ and provided the basis for the recommendation of a third primary mRNA vaccine dose.^1^ Second, participants who reported a high‐risk exposure in the 14 days before illness onset demonstrated lower three‐dose VE during the Omicron predominant period compared with overall VE during this time. These results are consistent with previous studies, including an analysis of data from the US Flu VE Network during the Delta‐predominant period.^6^, ^28^, ^29^ Third, the proportion of participants reporting previous laboratory confirmed SARS‐CoV‐2 infection was higher when the Omicron variant predominated than when the Delta variant predominated. However, we were unable to evaluate the impact of time since prior infection on VE due to small sample sizes. In contrast, prior studies have demonstrated increased protection among persons with prior SARS‐CoV‐2 infection history.^30^, ^31^

This investigation is subject to at least six limitations. First, small sample sizes limited our ability to evaluate VE by certain subgroups. Differences between two‐ and three‐dose mRNA VE by vaccine product, age group, and underlying medical conditions have been reported from studies including larger numbers of patients or medical encounters.^3^, ^5^, ^11^, ^12^, ^13^, ^14^, ^15^ Second, adolescents and children were not included in this analysis due to lower proportion of enrollment than in typical influenza seasons and lower percent vaccinated. Third, because of recent authorization of a booster dose for adults, waning of three‐dose VE could not be assessed. Waning effectiveness of a booster dose against COVID‐19 associated emergency department or urgent care visits has been reported elsewhere, though the study population may have differed to a certain extent from that of the US Flu VE Network.^5^ Fourth, with active enrollment, persons consenting to participate may differ from all patients in ways that may affect VE estimates, such as different healthcare‐seeking behaviors among vaccinated and unvaccinated persons.^10^ Vaccinated SARS‐CoV‐2 positive patients may have been more likely than unvaccinated cases to participate in this study. Fifth, Delta versus Omicron misclassification among the subset of infections without sequencing results is possible. Finally, increased use of at‐home testing may result in changes in healthcare seeking behavior and potential biases for VE studies, which requires further examination.

VE studies that rely on active enrollment of patients meeting clinical criteria for acute respiratory illness may contribute to ongoing monitoring of effectiveness of current and future COVID‐19 vaccines.^8^ Studies in this outpatient setting also contribute to understanding vaccine protection against a spectrum of illness, adding effectiveness against symptomatic illness in outpatient settings to published inpatient, emergency department, and urgent care estimates for moderately severe and severe COVID‐19. Systematic testing of outpatients presenting with CLI has the potential to identify SARS‐CoV‐2 positive cases and collect vaccination histories that may not be available from analyses of electronic medical records, especially as SARS‐CoV‐2 testing for persons with symptomatic illness becomes less frequent.^32^ As SARS‐CoV‐2 viruses evolve and COVID‐19 continues to cause substantial respiratory illness, systematic testing for respiratory illnesses including COVID‐19 and influenza will be important to evaluate effectiveness of COVID‐19 vaccines and immunization schedules.

## CONFLICT OF INTEREST

Ana Florea reports unrelated institutional grant support for research from Gilead, GlaxoSmithKline, Moderna, and Pfizer. Carlos G. Grijalva reports consulting fees from Merck, Pfizer, and Sanofi Pasteur, and institutional grant support from the Agency for Health Care Research and Quality, Campbell Alliance/Syneos Health, the Food and Drug Administration, and the National Institutes of Health. Emily T. Martin reports institutional grant support from Merck. Arnold S. Monto reports personal fees from Sanofi and non‐financial support from Seqirus. Mary Patricia Nowalk reports unrelated institutional grant support and personal fees from Merck Sharp & Dohme and institutional investigator‐initiated grant support from Sanofi Pasteur. Sara Y. Tartof reports unrelated institutional grant support from Pfizer and GlaxoSmithKline. No other potential conflicts of interest were disclosed.

## AUTHOR CONTRIBUTIONS

**Sara Kim:** Conceptualization; formal analysis; project administration. **Jessie Chung:** Conceptualization; data curation; methodology. **Keipp Talbot:** Data curation. **Carlos Grijalva:** Data curation. **Karen Wernli:** Data curation. **Erika Kiniry:** Data curation. **Emily Martin:** Data curation. **Arnold Monto:** Data curation. **Edward Belongia:** Data curation. **Huong Q. McLean:** Data curation. **Manjusha Gaglani:** Data curation. **Mufaddal Mamawala:** Data curation. **Mary Patricia Nowalk:** Data curation. **Krissy Moehling:** Data curation. **Sara Tartof:** Data curation. **Ana Florea:** Data curation. **Justin Lee:** Data curation; methodology. **Mark Tenforde:** Conceptualization; methodology. **Manish Patel:** Conceptualization; supervision. **Brendan Flannery:** Conceptualization; methodology; supervision.

## DISCLAIMERS

The findings and conclusions in this report are those of the authors and do not necessarily represent the official position of the Centers for Disease Control and Prevention. Vaccination data from Pennsylvania were supplied by the Bureau of Health Statistics & Registries, Pennsylvania Department of Health, Harrisburg, Pennsylvania. The Pennsylvania Department of Health specifically disclaims responsibility for any analyses, interpretations, or conclusions.

### PEER REVIEW

The peer review history for this article is available at https://publons.com/publon/10.1111/irv.13029.

## Data Availability

Data are available by request from the authors.

## Footnotes

* See 45 C.F.R. part 46; 21 C.F.R part 56
